# A Pilot Study: Adaptation Phase of the PROMIS Women Education Program—Promoting Cervical Cancer Prevention Methods Among Muslim Women in Virginia

**DOI:** 10.1002/cam4.71296

**Published:** 2025-10-10

**Authors:** Asmaa Namoos, NourEldin Abosamak, Vanessa Sheppard

**Affiliations:** ^1^ School of Medicine Virginia Commonwealth University Richmond Virginia USA

**Keywords:** cervical cancer prevention, community engagement, health disparities, health education programs, HPV vaccination, Muslim women

## Abstract

**Purpose:**

The purpose of this comprehensive research project is to address the notable disparities in cervical cancer prevention experienced by Muslim women in Virginia, compared with non‐Muslim women. Low participation in prevention and control activities, such as cervical cancer screening and HPV vaccination, often leads to their diagnosis with late‐stage cervical cancer. The long‐term research goal is to develop a culturally appropriate and religiously adapted intervention program to promote cervical cancer screening and prevention among Muslim women. Driven by an integrative conceptual model, the primary aim is to adapt existing evidence‐based educational materials to create a religiously adapted and culturally appropriate intervention program to improve cancer screening rates among Muslim women in the U.S.

**Methods:**

The study adapted existing evidence‐based educational materials to fit religious and cultural contexts, facilitated through focus group sessions with 10 Muslim women aged 18 and older. Additionally, interviews with five Muslim religious leaders provided feedback on the materials. The PEN‐3 model was employed to categorize and analyze cultural factors influencing health behaviors.

**Results:**

Preliminary findings from the thematic analysis, structured around the three domains of cultural identity, relationships, and expectations, and cultural empowerment, indicated a strong positive reception and increased awareness among participants. Key themes identified include the importance of culturally sensitive health messages, the influential role of community leaders, and the need for educational materials that consider cultural and gender dynamics. Data saturation was reached after the second focus group session, as no new themes emerged in participating discussions. Additionally, the Community Advisory Board (CAB) actively participated in the refinement process by reviewing the educational materials alongside the research team, ensuring that the content was culturally appropriate and aligned with community needs. Participants demonstrated a heightened readiness to engage in preventive behaviors, highlighting the effectiveness of a culturally tailored educational approach.

## 1 Background

Cervical cancer is a significant global health burden, ranking as the fourth most common cancer among women, with an estimated 660,000 new cases and 350,000 deaths in 2022 [1]. In the United States, despite the effectiveness of screening and HPV vaccination programs in reducing incidence and mortality, the disease continues to disproportionately affect underprivileged populations [2]. Studies confirm that routine Pap smears and HPV vaccination significantly lower cervical cancer incidence and mortality rates [3], yet disparities persist due to barriers in healthcare access, financial constraints, and cultural influences.

Despite advancements in healthcare, disparities in cervical cancer diagnosis and treatment among minority populations in the U.S. remain a pressing concern [4]. Black and Hispanic women face higher incidence and mortality rates [5], with contributing factors such as limited insurance coverage and the high costs of screening [6]. A study from North Carolina reported that 61% of individuals aged 25 to 64 were not up to date with screenings, highlighting the impact of socioeconomic barriers [7]. Additionally, cultural, linguistic, and trust‐related factors further hinder screening participation. For example, research conducted in Chicago found that Muslim women, despite recognizing the importance of Pap tests, often experience religious and cultural influences that negatively affect their health‐seeking behaviors [8].

The lack of data on religious affiliations in U.S. Cancer Registries complicates the provision of tailored healthcare services to religiously conservative communities. In Virginia, comparative studies between Muslim and non‐Muslim women have shown that Muslim women frequently seek care at later stages of the disease and are less likely to have insurance coverage [9]. These findings, alongside in‐depth interviews and surveys conducted by Namoos et al., highlight a significant knowledge gap about Pap tests and the necessity for culturally and religiously sensitive educational programs. This research identified key themes such as cultural influences, misconceptions, and language barriers, which interact complexly with the U.S. healthcare system [10].

Despite the availability of services from providers like Planned Parenthood and community health centers, there remain gaps in care, particularly for socially conservative groups like Muslim women, who often lack access to culturally sensitive care. While there have been numerous initiatives tailored to Muslim communities in the U.S. for breast cancer screening, similar efforts are noticeably absent in cervical cancer prevention [10, 11]. This gap underscores a significant need for healthcare strategies that respect and address the unique challenges faced by Muslim women.

In response to these disparities, several specialized programs have been developed, leveraging a blend of international and local insights. Programs such as CervixCheck, AMIGAS, and MARHABA have been tailored to meet the cultural sensitivities and specific needs of minority communities, including Muslim Americans. These programs employ unique methods like spiritual messaging, culturally adapted educational materials, and community‐focused health education to improve cancer screening rates [12, 13]. The current study aims to adapt existing evidence‐based educational materials to create a religiously adapted and culturally appropriate intervention program.

## 2 Methods

### 2.1 Study Design

The study utilized a structured approach that began with two focus group sessions involving 10 Muslim women aged 18 or older. The initial session focused on selecting and defining educational targets and content. In the second session, the group worked with researchers to refine these materials. Insights from these discussions were critical in finalizing our intervention's educational materials. Additionally, feedback from individual interviews with five Muslim religious leaders from our Community Advisory Board (CAB) played a crucial role in shaping the development process and guiding the intervention's direction.

A convenient sampling approach was used to recruit participants due to the practical constraints of accessing a hard‐to‐reach population. While purposive sampling was considered, it was not feasible due to time limitations and recruitment challenges within the Muslim community. To mitigate potential biases, efforts were made to include a diverse range of participants based on age, education, and religious backgrounds. Additionally, recruitment was conducted across multiple community settings, such as mosques and cultural centers, to enhance representativeness. Participants included self‐identified Muslim women aged 18 and older who were fluent in English and capable of providing informed consent.

**FIGURE 1:**
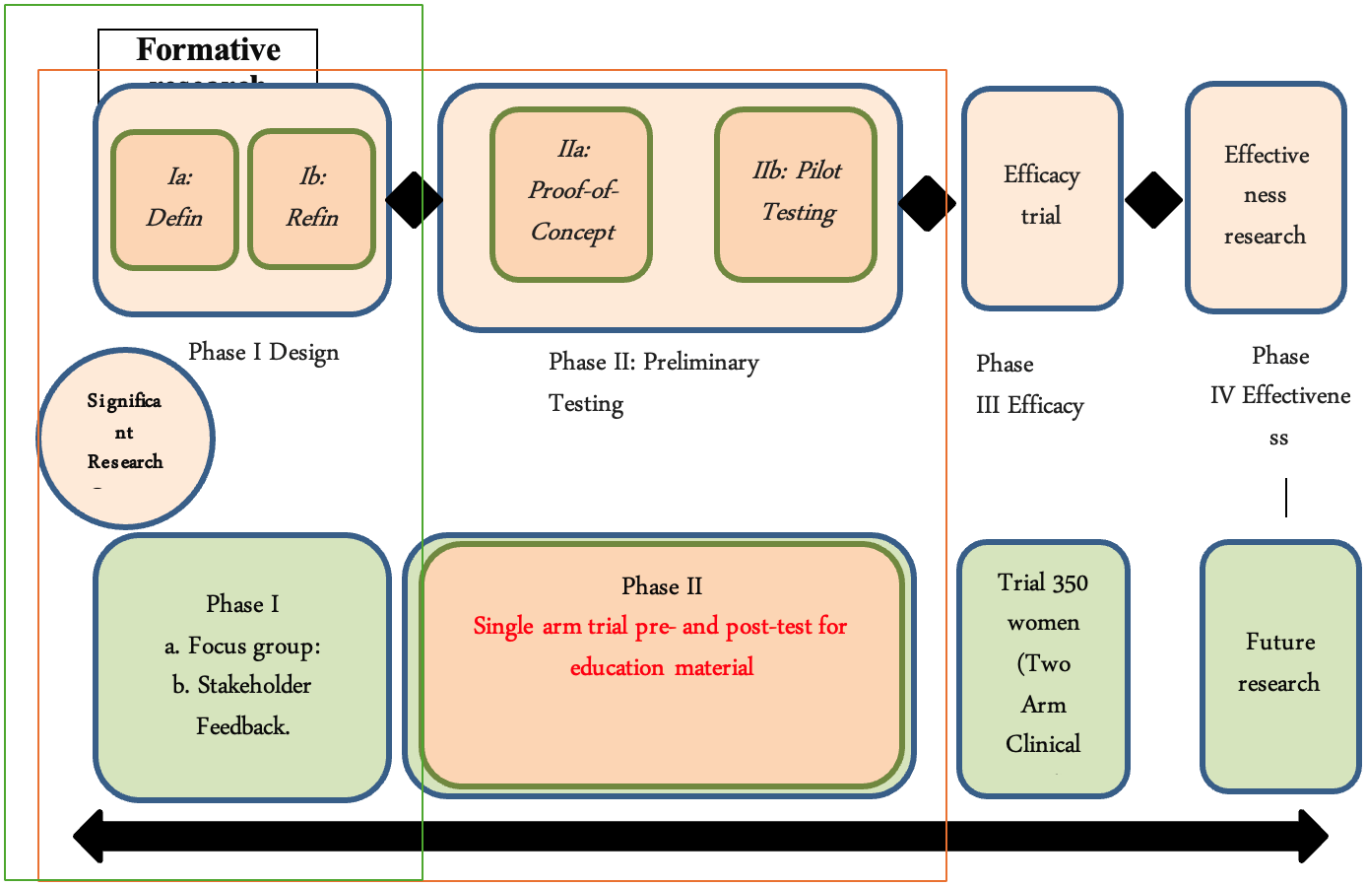
The ORBIT model for developing behavioral treatment for chronic disease.

The educational program was developed using the Obesity‐Related Behavioral Intervention Trials (ORBIT) model (Figure 1), which guided the tailored approach to behavioral treatments for chronic diseases. The Obesity‐Related Behavioral Intervention Trials (ORBIT) model provided a structured framework for systematically developing and refining the educational intervention in this study. This model was instrumental in guiding our research through a phased approach that incorporated community feedback and behavioral theories. In Phase I, formative research was conducted to assess the specific needs of the Muslim community regarding cervical cancer prevention. Focus group discussions were reviewed by the Community Advisory Board (CAB) to ensure cultural and religious appropriateness. Insights gained from these sessions were further analyzed using the PEN‐3 Model, which allowed for a deeper exploration of cultural factors influencing health behaviors. In Phase II, the Health Belief Model (HBM) was integrated to shape the educational content, ensuring that the materials aligned with participants' health perceptions and motivations. This structured adaptation of the ORBIT model facilitated the development of a culturally sensitive and evidence‐based intervention, ensuring that the program resonated with the target population and effectively addressed barriers to cervical cancer prevention.

#### 2.1.1 Research Setting and Community‐Engaged Framework

This project engaged Imams at mosques in and around Richmond, VA, utilizing a Community Action Model (CAM) to involve community members in every stage—from design to evaluation—of community action plans aimed at eliminating cervical cancer disparities. Over a 12‐month planning period, we focused on building community capacity, fostering partnerships, and establishing networks of community volunteers. We built strong partnerships between Virginia Commonwealth University and the Islamic Center of Virginia. This involved setting up a CAB and engaging Community Peer Researchers. The CABs, which included both Sunni and Shia religious leaders, played a crucial role in bridging trust gaps within the Muslim community and guided the CBPR efforts.

##### 2.1.1.1 Engagement and Material Adaptation

We engaged two women as peer researchers, well‐versed in community norms and technology, to navigate cultural sensitivities and maximize outreach among Muslim women. Additionally, we adapted existing evidence‐based educational materials to align with the cultural and religious nuances of the target community, culminating in the development of a culturally appropriate intervention program.

### 2.2 Consenting Process and Ethical Considerations

Our study team, including peer researchers, recruited Muslim women at the Islamic Center by distributing flyers and screening for eligibility. During the consenting process, the team provided a detailed explanation of the study's goals, potential risks, benefits, confidentiality measures, and incentives. Ethical approval was granted by the Institutional Review Board (IRB). All participants gave informed consent, and their confidentiality was ensured by pseudonyms and secure data storage.

### 2.3 Data Collection

Data collection occurred in a private room at a Mosque, facilitated by the Islamic Center of Virginia to ensure a comfortable setting for participants. We conducted two focus group sessions with the same group. The first session focused on selecting educational targets and content related to cervical cancer awareness. In the follow‐up session a week later, participants reviewed and provided feedback on the formatting and usability of the educational materials. Data was securely stored in the secure IT system, accessible only to authorized personnel, ensuring confidentiality and ethical data management.

### 2.4 Description of the PROMIS Women Education Program

The PROMIS Women Education Program aims to enhance cervical cancer prevention knowledge among Muslim women through culturally appropriate and religiously adapted educational materials to increase knowledge, self‐efficacy, and positive intentions towards screening and vaccination. The program draws on insights from various sources, including the MARHABA, CervixCheck, and AMIGAS projects [12, 13, 14].

### 2.5 Workshop for Developing Educational Material

The development process for the educational materials involved several key steps: First, the research team selected and summarized existing education programs. Next, focus groups comprising Muslim women chose culturally appropriate materials and recommended incorporating Quranic statements. Based on this feedback, the research team adjusted and refined the materials. These refined materials were then compiled and reviewed by both the research team and the CAB, which further suggested the inclusion of spiritual statements from the Quran. Throughout, peer researchers facilitated the focus group sessions, creating a supportive environment that encouraged open dialogue and thorough feedback.

### 2.6 Data Analysis

Data analysis utilized thematic analysis to scrutinize audio recordings and field notes. Iterative coding was used to identify key themes related to cultural, religious, and healthcare factors affecting cervical cancer prevention, supported by descriptive statistics in R Studio. Additionally, we employed the PEN‐3 model to examine cultural influences on health behaviors. This model assesses three dimensions—Cultural Identity, Relationships and Expectations, and Cultural Empowerment—enabling a culturally sensitive analysis of participant experiences.

### 2.7 Data Saturation

We monitored data saturation by regularly reviewing collected data and analyzing emerging themes. After the second focus group session, we observed that no new themes were emerging, indicating that data saturation had been reached.

Overall, our qualitative research design, participant selection, data collection methods, and rigorous analysis allowed us to comprehensively explore and address cervical cancer disparities within the Muslim community.

### 2.8 Ethical Considerations

This study received ethical approval from the Virginia Commonwealth University Institutional Review Board (VCU IRB). All participants provided informed consent, and confidentiality was maintained throughout the study.

## 3 Results

### 3.1 Participant Demographics

Our study involved 10 Muslim women aged between 28 and 78, representing diverse backgrounds in education, employment, and language proficiency. Race/ethnicity distribution included White (40%), Black (30%), and Middle Eastern (30%) participants. Regarding marital status, the majority were married (70%), with 10% each being single, divorced, or widowed.

Educational backgrounds varied, with 10% having only a high school education, 40% completing some college, another 40% earning a college degree, and 10% holding postgraduate qualifications. In terms of employment status, 50% were employed, 40% were not employed, and 10% were retired. Language proficiency was also assessed, with 70% of participants being proficient in English, 20% at an intermediate level, and 10% with only basic proficiency. Arabic was the primary home language among participants, though many demonstrated bilingual proficiency (Table 1).

**TABLE 1:** Demographics of the study participants.

Category	Sub‐category	Number of participants (*n* = 10)	Proportion (%)
Race/ethnicity	White	4	40
	Black	3	30
	Middle Eastern	3	30
	Hispanic	0	0
Marital status	Single	1	10
	Married	7	70
	Divorced	1	10
	Widow	1	10
Education level	High School	2	10
	Some College	4	40
	College Graduate	4	40
	Postgraduate	1	10
Employment status	Employed	5	50
	Not Employed	4	40
	Retired	1	10
English proficiency	Basic	1	10
	Intermediate	2	20
	Proficient	7	70

### 3.2 The PEN‐3 Cultural Model Domains: Relationships and Expectations/Cultural Empowerment Analysis (Table 2)

**TABLE 2:** PEN‐3 cultural model domains.

PEN‐3	Positive	Existential	Negative
Perceptions	Appreciation for culturally sensitive health messages. Health messages integrated with religious values Positive perceptions: “We, as Muslim women, seek knowledge and empowerment in our health journey. By adapting education to respect our culture and faith, we confidently join hands in preventing cervical cancer, embracing our well‐being with wisdom and trust” (P4)	Need for health materials to reflect cultural identity. Alignment of health communication with cultural and religious beliefs. “I care deeply about our health as conservative culture. Preventing cervical cancer is more than just taking care of our bodies; it's also about taking care of our spirits. It helps us protect our lives in a way that fits with our culture and beliefs” (P7)	Lack of cultural sensitivity in materials. Challenges in addressing cultural taboos respectfully. “We need educational materials on health that speak to us as Muslim women. Let's change the way we learn about cervical cancer prevention, so it respects who we are and what we believe in. It's time for information that understands and supports every woman” (P3)
Enablers	Narratives featuring empowered women. Inclusion of guidance for male family members in decision‐making “Let's create educational materials that speak to every family member. By learning together from brochures and guides about cervical cancer prevention, we ensure that our fathers, brothers, and husbands are involved and informed, enhancing our family's knowledge and unity in making health decisions” (P5)	Community power “Our community leaders guide us in making good choices. When they share knowledge about cervical cancer prevention, it helps us all. Let's listen to them and learn, so we can make smart health decisions for ourselves and our families” (P10)	Difficulties with medical terminology comprehension. Lack of consideration for cultural and gender dynamics in health decision‐making: “Sometimes medical words are hard to understand, and they don't always fit with our way of life or respect who we are—men and women in our community” (P3)
Nurturers	Respect for family traditions in health education. Community norms and religious endorsements in health communication: “The ad featuring a woman with a hijab in the Middle East is a powerful representation of our traditions. It resonates with our culture, and the presence of a woman of color with a hijab reflects our values and identity” (P1)	Culturally sensitive visuals in health materials‐ Inclusion of local stories and testimonials to make information relatable and trustworthy “Including local stories or testimonials, as seen in the MARHABA Trial, makes the information more relatable and trustworthy” (P8) “Effectively conveys the importance of culturally sensitive visuals and the inclusion of local stories and testimonials in health materials. It highlights how these elements enhance the relatability and trustworthiness of the information, as demonstrated in the MARHABA Trial” (P8)	Influence of harmful cultural beliefs on health practices‐ Barriers in confronting myths and taboos in health education “Changing the vague imagery in these ads could engage the reader more and enhance their understanding of cervical cancer” (P2) “Respect for our community norms is important. The visuals in some of these materials need to be more modest to be acceptable in our community” (P1)

#### 3.2.1 Perceptions

##### 3.2.1.1 Positive Perceptions

The findings suggest that Muslim women value health education that is culturally and religiously sensitive. This desire for empowerment and knowledge in health, specifically in cervical cancer prevention, is evident in Participant P4's statement: “We, as Muslim women, seek knowledge and empowerment in our health journey. By adapting education to respect our culture and faith, we confidently join hands in preventing cervical cancer, embracing our well‐being with wisdom and trust.”

##### 3.2.1.2 Existential Perceptions

Participant P7's quote highlights the importance of aligning health messages with conservative cultural values and spiritual beliefs. This holistic approach to health, where physical and spiritual well‐being are interconnected, is captured in their words: “I care deeply about our health as a conservative culture. Preventing cervical cancer is more than just taking care of our bodies; it's also about taking care of our spirits. It helps us protect our lives in a way that fits with our culture and beliefs.”

##### 3.2.1.3 Negative Perceptions

The gap in current health communication strategies is pointed out by Participant P3. They emphasize the need for culturally sensitive educational materials, as stated: “We need educational materials on health that speak to us as Muslim women. Let's change the way we learn about cervical cancer prevention, so it respects who we are and what we believe in. It's time for information that understands and supports every woman.”

These quotes from the participants effectively underline the importance of culturally and religiously adapted health education, reflecting a nuanced understanding of their specific needs and values in health communication.

#### 3.2.2 Enablers

##### 3.2.2.1 Positive Enablers

The participant's quote reflects the importance of inclusive educational materials that engage all family members, including male relatives, in health decisions. As expressed by Participant P5: “Let's create educational materials that speak to every family member. By learning together from brochures and guides about cervical cancer prevention, we ensure that our fathers, brothers, and husbands are involved and informed, enhancing our family's knowledge and unity in making health decisions.” This highlights the value of collective learning and decision‐making in family units, emphasizing the role of men in supporting and understanding women's health issues.

##### 3.2.2.2 Existential Enablers

Participant P10's statement underscores the influential role of community leaders in disseminating health information. The quote: “Our community leaders guide us in making good choices. When they share knowledge about cervical cancer prevention, it helps us all. Let's listen to them and learn, so we can make smart health decisions for ourselves and our families,” suggests that community leaders are pivotal in educating and guiding the community towards better health practices. This reflects the community's trust and reliance on their leaders for guidance in making informed health choices.

##### 3.2.2.3 Negative Enablers

The difficulties in understanding medical terminology and the lack of consideration for cultural and gender dynamics in health decision‐making are significant barriers. Participant P3 voices this concern: “Sometimes medical words are hard to understand, and they don't always fit with our way of life or respect who we are – men and women in our community.” This highlights the need for health communication that is not only linguistically accessible but also culturally relevant and sensitive to gender dynamics within the community.

These findings emphasize the necessity for health education that is culturally sensitive, inclusive, and community‐oriented. By addressing these enablers and barriers, health communication can be more effective in reaching and engaging the target audience, leading to better health outcomes.

#### 3.2.3 Nurturers

##### 3.2.3.1 Positive Nurturers

Participant P1's comment emphasizes the importance of respecting family traditions and community norms in health education. The quote: “The ad featuring a woman with a hijab in the Middle East is a powerful representation of our traditions. It resonates with our culture, and the presence of a woman of color with a hijab reflects our values and identity,” illustrates how culturally relevant visuals, such as a woman wearing a hijab, positively impact the community's reception of health messages. This approach fosters a sense of identification and respect for cultural and religious values.

##### 3.2.3.2 Existential Nurturers

The existential aspect is underlined by Participant P8, who recognizes the value of incorporating local stories and testimonials in health materials. Their statement: “Including local stories or testimonials, as seen in the MARHABA Trial, makes the information more relatable and trustworthy,” highlights how personal narratives and culturally sensitive visuals enhance the relevance and credibility of health information, making it more engaging and effective for the community.

##### 3.2.3.3 Negative Nurturers

On the other hand, Participant P2 and P1 point out the challenges related to harmful cultural beliefs and the difficulty in confronting myths and taboos in health education. P2's observation: “Changing the vague imagery in these ads could engage the reader more and enhance their understanding of cervical cancer,” along with P1's remark: “Respect for our community norms is important. The visuals in some of these materials need to be more modest to be acceptable in our community.” reflect the need for careful consideration of cultural sensitivities and norms in health communication. This includes addressing myths and taboos delicately and ensuring that visual content is both informative and culturally appropriate.

These insights suggest that effective health education for cervical cancer prevention should be deeply rooted in cultural understanding and respect for community norms.

### 3.3 Community Advisory Board Suggestions

The Community Advisory Board (CAB) provided critical feedback to refine the PROMIS Women Education Program materials. They commended the integration of evidence‐based content but recommended incorporating up‐to‐date statistics to enhance relevance for the target audience. To further strengthen cultural appropriateness, the CAB advised additional consultations with community leaders to ensure that all cultural aspects were accurately represented.

Beyond addressing cultural sensitivities, the CAB emphasized the need to celebrate positive cultural practices that support women's health. Regarding readability, they found the materials informative but suggested simplifying complex medical terminology and adding visual aids to improve comprehension. Finally, they underscored the importance of multilingual accessibility to reach a broader audience and maximize impact.

### 3.4 Formative Research Outcome

Based on the themes, recommendations, and input from the CAB, we have developed a comprehensive study guidebook, accompanied by three informative videos and three educational brochures. Informed by the Health Belief Model Theory as the theoretical underpinning, our comprehensive study guidebook, informative videos, and educational brochures have been crafted to enhance individual‐level knowledge about cervical cancer and HPV vaccination. Each component of these materials is designed to address specific aspects of the Health Belief Model—perceived susceptibility, perceived severity, perceived benefits, perceived barriers, and self‐efficacy. The guidebook provides detailed information in an easy‐to‐understand format, breaking down complex medical terms to improve comprehension. The videos, featuring culturally sensitive visuals and narratives, aim to increase awareness of the risks and seriousness of cervical cancer, while also highlighting the benefits and accessibility of preventive measures like the Pap smear test and HPV vaccination. The brochures, enriched with testimonials and local narratives, aim at reducing perceived barriers and enhancing self‐efficacy by empowering Muslim women with the knowledge and confidence to take proactive health actions. These materials, respectful of cultural and religious values, not only educate but also encourage positive health behavior changes, such as important cues to action, etc., in line with the Health Belief Model, thus ensuring a more effective and culturally attuned intervention program for the Muslim community.

## 4 Discussion

The study aimed to curate a culturally appropriate and religiously tailored educational program aimed at improving cervical cancer prevention and screening activities among Muslim women in the U.S. The analysis of the PEN‐3 cultural model highlights the significant potential impact of culturally and religiously sensitive health education for Muslim women on cervical cancer prevention. Muslim women appreciate health education efforts that respect their beliefs, empowering them to take proactive steps in their health journey. In the study, they emphasized the need for health messages that align with both their physical and spiritual well‐being, reflecting their conservative cultural values. This aligns with the existing literature, which suggests that culturally tailored interventions are more likely to engage target populations and improve health outcomes [15, 16].

However, the study reveals a gap in current health communication, with educational materials often lacking cultural relevance. There is a demand for inclusive materials that engage entire families, including male relatives, in health decisions, and for utilizing community leaders' influential roles in health guidance. Despite these positive aspects, barriers such as complex medical terminology and culturally insensitive content showed up in the study, mirroring recommendations and findings from the literature, preventing effective communication [17, 18].

To be effective, participants expressed that health education must respect cultural norms, use relatable visuals, and address harmful beliefs and taboos in a way that resonates with the community. The findings highlight the need for health education that is not only informative but also deeply rooted in cultural understanding to improve health outcomes.

The findings align with existing research on the effectiveness of culturally tailored health interventions for minority populations, such as the MARHABA, CervixCheck, and AMIGAS projects. This study adds to the literature by addressing the specific needs of Muslim women, particularly the importance of incorporating religious and cultural values into health education.

The feedback from the CAB emphasized the importance of celebrating rather than merely accommodating cultural practices. The adaptation of educational materials to include culturally appropriate visuals and local stories was well‐received and contributed to a more relatable and effective intervention. Our approach leverages cultural strengths and fosters a more supportive environment for health education.

### 4.1 Limitations and Future Directions

As researchers, our background and experiences could impact the research process and findings. We acknowledged our potential biases and worked to minimize our influence. Our commitment to cultural sensitivity and community collaboration helped mitigate potential biases. Throughout the research process, we engaged in reflexivity by critically examining our own beliefs and assumptions. Regular team discussions helped us navigate potential biases and be open to diverse perspectives from the participants.

However, the study had several limitations. The small sample size and regional focus restrict the generalizability of the findings. Additionally, participants were limited to English‐speaking Muslim women, which may not fully capture the experiences of non‐English speakers or those from different regions of the U.S. Future research should involve larger, more diverse samples, including individuals with limited English proficiency, and explore the long‐term impact of culturally tailored materials. Further, the reliance on self‐reported data may introduce response bias, which could be addressed in future studies through mixed‐method approaches.

The use of convenience sampling presents another limitation, as it may introduce selection bias and limit external validity. However, this approach was necessary given the logistical challenges of reaching the target population. Despite these constraints, steps were taken to minimize bias by ensuring diverse representation within the sample and engaging multiple recruitment sites. Future studies could explore the use of purposive sampling or mixed‐method approaches to strengthen participant selection and improve external validity.

### 4.2 Implications for Future Program Development

While this study demonstrates that culturally and religiously adapted educational materials can improve cervical cancer prevention knowledge and practices among Muslim women, further iterations of the program should consider integrating technology and digital health tools. Mobile health (mHealth) applications, interactive webinars, and culturally adapted telehealth consultations could enhance accessibility and engagement, particularly for younger and digitally connected populations. Future interventions may also incorporate AI to provide real‐time health guidance in multiple languages, making the program more inclusive.

## 5 Conclusion

This study highlights the value of culturally and religiously tailored education in improving awareness of cervical cancer prevention among Muslim women. While the findings suggest that these adaptations help foster engagement, further research is needed to understand their long‐term impact on health behaviors.

This paper has paved the way for Phase II, which will focus on intervention sessions aimed at assessing how these educational materials influence preventive actions.

By addressing cultural sensitivities and engaging community leaders, we can create more effective and respectful health interventions. Ongoing efforts to refine and expand these programs will be crucial in reducing health disparities and promoting better health outcomes for underserved populations. Future research should explore how integrating technology‐based interventions can further enhance engagement and impact.

## Author Contributions

**Asmaa Namoos:** conceptualization (lead), data curation (lead), formal analysis (lead), investigation (lead), methodology (lead), project administration (lead), resources (supporting), software (lead), supervision (lead), validation (lead), visualization (lead), writing – original draft (lead), writing – review and editing (lead). **NourEldin Abosamak:** conceptualization (supporting), data curation (supporting), formal analysis (supporting), funding acquisition (supporting), investigation (supporting), methodology (lead), supervision (supporting), validation (supporting), writing – original draft (lead). **Vanessa Sheppard:** conceptualization (supporting), data curation (supporting), formal analysis (supporting), funding acquisition (supporting), investigation (supporting), methodology (supporting), project administration (lead), resources (lead), software (lead), supervision (lead), validation (lead), visualization (lead), writing – original draft (supporting), writing – review and editing (lead).

## Conflicts of Interest

The authors declare no conflicts of interest.

## Data Availability

The data supporting the findings of this study will be available upon reasonable request. Interested researchers must complete the required forms in accordance with the Virginia Commonwealth University Institutional Review Board (VCU IRB) requirements to gain access to the data. Requests can be directed to the corresponding author.
